# Ankylosing spondylitis patients at risk of poor radiographic outcome show diminishing spinal radiographic progression during long-term treatment with TNF-α inhibitors

**DOI:** 10.1371/journal.pone.0177231

**Published:** 2017-06-22

**Authors:** Fiona Maas, Suzanne Arends, Freke R. Wink, Reinhard Bos, Hendrika Bootsma, Elisabeth Brouwer, Anneke Spoorenberg

**Affiliations:** 1 Rheumatology and Clinical Immunology, University Medical Center Groningen, University of Groningen, Groningen, The Netherlands; 2 Rheumatology, Medical Center Leeuwarden, Leeuwarden, The Netherlands; University of Birmingham, UNITED KINGDOM

## Abstract

**Objective:**

To investigate the influence of patient characteristics on the course of spinal radiographic progression in a large prospective longitudinal cohort study of ankylosing spondylitis (AS) patients treated long-term with TNF-α inhibitors.

**Methods:**

Consecutive patients from the Groningen Leeuwarden AS (GLAS) cohort starting TNF-α inhibitors with spinal radiographs at least available at baseline and 6 years of follow-up were included. Radiographs were scored using mSASSS by two independent readers. Generalized estimating equations (GEE) were used to explore the associations between baseline characteristics and spinal radiographic progression. The course of radiographic progression in patients with and without risk factors for poor radiographic outcome was investigated using different time models (linear and non-linear). Single linear imputation was used in case of missing radiographic data at the intermediate (2 or 4 years) follow-up visits.

**Results:**

80 AS patients were included with mean baseline mSASSS 8.7±13.3. Baseline syndesmophytes, male gender, older age, longer symptom duration, smoking, and higher BMI were significantly associated with more radiographic damage over time. GEE analysis in patients with these risk factors revealed that radiographic progression followed a non-linear course with mean mSASSS progression rates reducing from max. 2.8 units over 0–2 years to min. 0.9 units over 4–6 years. The GEE model revealed a linear course with overall very low progression (≤1 mSASSS units/2yrs) in patients without risk factors. Complete case analysis in 53 patients showed similar results.

**Conclusion:**

AS patients at risk of poor radiographic outcome showed the highest but diminishing spinal radiographic progression during long-term treatment with TNF-α inhibitors.

## Introduction

In view of the clinical evaluation of new potential biological therapies in axial spondyloarthritis (axSpA) including ankylosing spondylitis (AS), it is important to identify which patients are at risk for radiographic progression. In earlier studies, spinal radiographic progression was found to be associated with the presence of baseline syndesmophytes, male gender, older age, smoking, worse functional status, and higher disease activity at baseline.[1–7] Among these risk factors, the presence of baseline syndesmophytes is the strongest predictor.[5,6,8] In our previous analysis of 176 AS patients long-term treated with tumor necrosis factor-alpha (TNF-α) inhibitors, patients with baseline syndesmophytes showed a 4-fold higher progression rate than patients without syndesmophytes.[4] Furthermore, elevated C-reactive protein (CRP) was identified as a strong predictor (OR 4.7 in multivariable model) for the progression of non-radiographic axSpA to AS based on the modified New York criteria.[9]

In addition to baseline risk factors, previous cohort studies in axSpA patients, mainly treated with non-steroidal anti-inflammatory drugs (NSAIDs), have demonstrated that spinal radiographic progression is associated with disease activity over time.[10,11] In the German Spondyloarthritis Inception Cohort (GESPIC), mean AS disease activity scale (ASDAS), erythrocyte sedimentation rate (ESR), and CRP over 2 years were significantly associated with spinal radiographic progression during these 2 years.[10] In the historical Outcomes in AS International Study (OASIS), a longitudinal relationship was found between spinal radiographic progression and assessments of disease activity over a follow-up period up to 12 years. Bath AS disease activity index (BASDAI), ASDAS, and CRP at the start of a 2-year time interval were significantly associated with radiographic progression during the next 2 years.[11]

Based on the multiple reported associations between disease activity over time and radiographic progression, we hypothesized that prolonged inhibition of disease activity could eventually lead to less spinal radiographic progression over time. In our recent study using longitudinal modeling of spinal radiographic progression in AS patients treated with TNF-α inhibitors, a deflection from a linear course with significantly decreasing progression rates was found at the group level after more than 4 years of follow-up (estimated mean progression rates reduced from 1.7 over 0–2 years to 1.0 over 4–6 years).[12]. Since individual progression rates were highly variable, it is important to explore the course of radiographic progression at individual patient level and to identify patient characteristics associated with this reduction in spinal radiographic progression. Therefore, the aim of the present study was to investigate the influence of patient characteristics on the course of spinal radiographic progression in AS patients treated long-term with TNF-α inhibitors.

## Methods

For the present study, we included consecutive outpatients from the Groningen Leeuwarden AS (GLAS) cohort who started treatment with TNF-α inhibitors between 2004 and 2009 and had spinal radiographs available at baseline and after 6 years of follow-up. Patient selection criteria and details about the study design have been described previously.[12] The GLAS cohort is a Dutch ongoing prospective longitudinal observational cohort study with a standardized assessment and management protocol. Included patients were 18 years or older, fulfilled the modified New York criteria for AS, and the ASAS criteria to start TNF-α inhibitors.[13] The GLAS cohort was approved by the local ethics committees of the Medical Center Leeuwarden (MCL) and the University Medical Center Groningen (UMCG). All patients provided written informed consent according to the Declaration of Helsinki.

### Patient characteristics

The following baseline characteristics were collected: gender, age, symptom duration, time since diagnosis, HLA-B27 status, body mass index (BMI), smoking status (no/previous smoker vs. current smoker), smoking duration (of current and previous smokers), use of NSAIDs (yes/no), use of disease-modifying antirheumatic drugs (DMARDs, yes/no), first TNF-α inhibitor (etanercept vs. infliximab/adalimumab), BASDAI, ASDAS, patient’s global assessment of disease activity (GDA), CRP level, and Bath AS functional index (BASFI).

### Radiological assessment

Radiographs of the cervical and lumbar spine were scored in chronological time order by two trained readers (FM and IE) using the modified Stoke AS spine score (mSASSS).[14,15] Radiographs were randomized and scored together with radiographs of AS patients who were not treated with TNF-α inhibitors from a historical cohort in order to avoid potential reader bias concerning the applied therapy. Patient characteristics were removed from the radiographs. Further details about the scoring method including inter-observer reliability have been described previously.[12]

### Statistical analysis

Spinal radiographic progression was evaluated using generalized estimating equations (GEE). GEE is a statistical technique used to investigate longitudinal relationships between variables. The advantage of GEE is that all available data at different time points (0, 2, 4, 6 year) are included in the model. GEE can correct for the fact that measurements over different time points are highly correlated within patients. In the present study, an exchangeable correlation structure was used.[3,4,12]

First, univariable GEE were used to explore the associations between patient characteristics at baseline and spinal radiographic damage over time. Multivariable GEE was used to identify the independent risk factors for radiographic progression.

Second, the course of spinal radiographic progression was investigated after stratifying the patients with and without risk factors for poor radiographic outcome. In case continuous variables were significantly associated with radiographic damage over time, stratification into subgroups was based on clinically relevant values or medians. In these subgroups, the course of spinal radiographic damage over time was investigated using linear and different non-linear time models (quadratic, cubic, square root, logarithmic, exponential); mSASSS was the independent variable and time the dependent variable. The time model with a significant contribution (p-value ≤0.05) and the lowest quasi-likelihood information criterion (QICC) represents the best model for the data. The mean mSASSS status scores at baseline and 2, 4 and 6 years and the mean 2-year progression rates were calculated based on the estimated intercept and regression coefficients obtained from this model.

The analyses were performed in patients with radiographic data available at baseline and after 6 years of follow-up. In case a patient had missing data at one or more intermediate follow-up visits (2 or 4 year), missing radiographic data was imputed using a single linear imputation technique. With this technique, an ongoing linear progression was assumed during the imputed time interval.[12] Additionally, complete case analysis was performed in patients with complete radiographic data at all 2-year time points. Statistical analysis was performed with IBM SPSS Statistics 22 (SPSS, Chicago, IL, USA).

## Results

In total, 98 AS patients from the GLAS cohort had 6 years of follow-up of which 80 had radiographic data available at baseline and at 6 years. These 80 patients had similar patient characteristics as the 18 patients without radiographic data (data not shown). Complete biannual radiographic data at all the intermediate time points (0, 2, 4, 6 years) were available for 53 (66%) patients. Baseline characteristics did not significantly differ between patients with and without complete radiographic data, except for the distribution of first prescribed TNF-α agent (S1 Table).

Almost all patients had high disease activity at baseline and stable, low disease activity over time after the start of TNF-α inhibitors (S1 Fig).

Of the 80 included patients, most started treatment with etanercept (n = 50), followed by both infliximab (n = 15) and adalimumab (n = 15). Fifty-five (69%) patients continued using their first TNF-α agent and 23 (29%) patients switched to another TNF-α agent during follow-up. Only 2 (2%) patients stopped TNF-α inhibition because of inefficacy. The mean duration of TNF-α inhibition was 5.4±1.2 years.

Included patients had a median mSASSS at baseline of 3.3 (IQR: 0.0–12.0), a mean mSASSS of 8.7±13.3, and 43 (54%) patients had one or more non-bridging or bridging syndesmophytes (Table 1).

**Table 1:** Baseline characteristics of included AS patients.

	Total group n = 80
**Male gender**	56 (70)
**Age (yrs)**	41.3 ± 10.5
**Symptom duration (yrs)**	14 (8–24)
**Time since diagnosis (yrs)**	5 (1–15)
**HLA-B27+**	62 (78)
**BMI (kg/m**^**2**^**)**	25.6 ± 3.8
**Current smoker**	28 (42)
**Smoking duration (yrs)**	13 (0–26)
**NSAID use**	69 (86)
**ASAS-NSAID index**	50 (25–100)
**DMARD use**	21 (26)
**First TNF-α inhibitor**	
Infliximab	50 (63)
Etanercept	15 (19)
Adalimumab	15 (19)
**BASDAI (0–10)**	6.0 ± 1.7
**ASDAS**_**CRP**_	3.8 ± 0.8
**Patient’s GDA (0–10)**	7 (5–8)
**CRP (mg/L)**	14 (7–23)
**ESR (mm/hr)**	21 (13–34)
**BASFI (0–10)**	5.6 (3.6–7.1)
**mSASSS (range 0–72) mean**	8.7 ± 13.3
**median**	3.3 (0.0–12.0)
**≥1 syndesmophyte**	43 (54)

Values are presented as number of patients (%), mean ± SD, or median (IQR).

AS: ankylosing spondylitis; HLA: human leukocyte antigen; BMI: body mass index; NSAID: non-steroidal anti-inflammatory drug; ASAS: Assessment of SpondyloArthritis international Society; DMARD: disease-modifying anti-rheumatic drug; BASDAI: Bath AS disease activity index; ASDAS: AS disease activity score; GDA: global disease activity; BASFI: Bath AS functional index; CRP: C-reactive protein; mSASSS: modified Stoke AS spine score.

### Patient characteristics associated with radiographic damage over time

Male gender, age, symptom duration, BMI, and baseline damage were significantly associated with spinal radiographic damage over time (Table 2). Similar result were found for patients using etanercept vs. infliximab or adalimumab. Multivariable GEE revealed that the presence of baseline syndesmophytes was the only independent predictor for radiographic progression.

**Table 2:** Associations between baseline characteristics and spinal radiographic damage over time.

	Total group (n = 80)	
	B (95% CI)	p-value
**Male gender**	8.87 (3.37–14.38)	**0.002**
**Age (yrs)**	0.70 (0.41–0.98)	**<0.001**
Age **≥**40 years	9.49 (3.53–15.45)	**0.002**
**Symptom duration (yrs)**	0.74 (0.36–1.11)	**<0.001**
Symptom **≥**10 years	12.29 (7.50–17.07)	**<0.001**
**Time since diagnosis (yrs)**	0.50 (-0.01–1.01)	0.056
**HLA-B27+**	0.23 (-7.37–7.82)	0.953
**Current smoker**	6.16 (-0.05–12.82)	0.070
**Smoking duration**	0.28 (-0.03–0.58)	0.072
**BMI (kg/m**^**2**^**)**	1.53 (0.41–2.64)	**0.007**
BMI ≥25 kg/m^2^	12.62 (4.85–20.40)	**0.001**
**NSAID use**	4.17 (3.30–11.63)	0.274
**DMARD use**	-2.38 (-9.70–4.95)	0.525
**First TNF-α inhibitor**†	-2.70 (-9.65–4.26)	0.447
**BASDAI (0–10)**	-0.59 (-2.17–0.98)	0.461
**ASDAS**_**CRP**_	1.91 (-2.30–6.11)	0.375
**Patient’s GDA (0–10)**	-0.33 (-1.59–0.94)	0.610
**CRP (mg/L)**	0.08 (-0.14–0.30)	0.456
**BASFI (0–10)**	0.85 (-0.44–2.14)	0.197
**mSASSS**	1.11 (1.05–1.17)	**<0.001**
**≥1 syndesmophyte**	18.33 (13.57–23.10)	**<0.001**

See Table 1 for abbreviations.

^†^Etanercept vs. infliximab/adalimumab

Complete case analysis in 53 AS patients with radiographic data available at all 2-year time points revealed similar results. Additionally, time since diagnosis and current smoking status reached statistical significance (p<0.05, S2 Table).

### Radiographic progression in patients with risk factors

**Fig 1:**
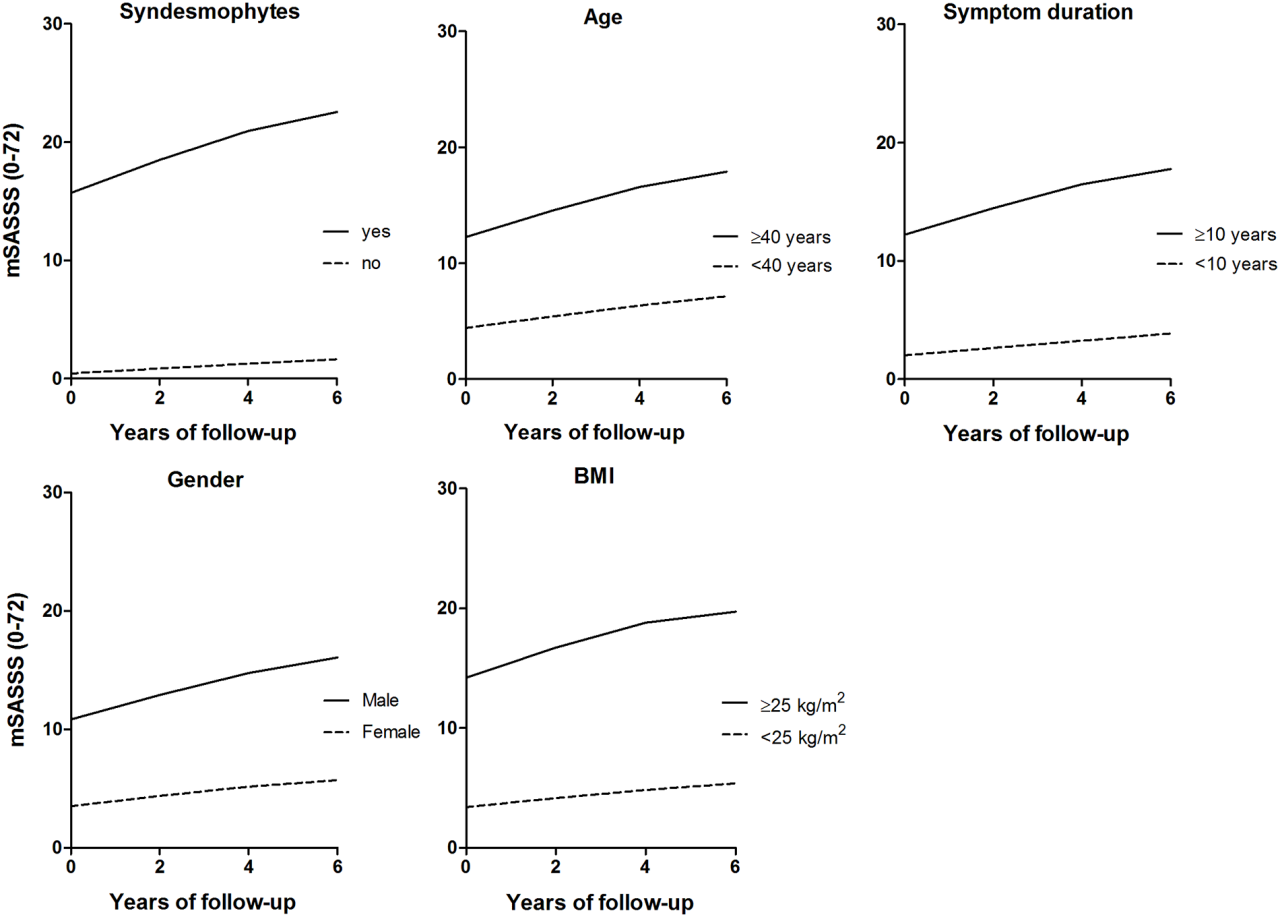
GEE estimated course of spinal radiographic progression in AS patients with 6 years of follow-up (n = 80), stratified for baseline risk factor.

Patients with baseline syndesmophytes, male gender, age ≥40 years, symptom duration ≥10 years, and BMI ≥25kg/m^2^ had higher baseline mSASSS and higher progression rates than patients without these characteristics (Table 3). Longitudinal modeling stratified for presence of these patient characteristics revealed that the course of spinal radiographic progression was non-linear with reducing progression rates over time (Table 3, Fig 1). The mean estimated progression rates reduced from maximal 2.8 mSASSS units over 0–2 years to minimal 0.9 mSASSS units over 4–6 years. The strongest deflection from a linear course was found in patients with baseline syndesmophytes and in patients with baseline BMI ≥25kg/m^2^ (Table 3).

**Table 3:** GEE estimated mean 2-year spinal radiographic progression rates, stratified for baseline risk factors.

		n	Course of progression	mSASSS progression rates		
				0–2 year (95% CI)	2–4 year (95% CI)	4–6 year (95% CI)
**Total group**		80	Non-linear	1.7 (1.1–2.3)	1.5 (0.8–2.3)	1.0 (-0.1–2.1)
**Syndesmophytes**	**Yes**	43	Non-linear	2.8 (1.9–3.7)	2.5 (1.3–3.6)	1.6 (-0.1–3.3)
	**No**	37	Linear	0.4 (0.2–0.7)	0.4 (0.2–0.7)	0.4 (0.2–0.7)
**Gender**	**Male**	56	Non-linear	2.1 (1.3–2.8)	1.9 (0.9–2.8)	1.3 (-0.1–2.7)
	**Female**	24	Linear	0.7 (0.3–1.2)	0.7 (0.3–1.2)	0.7 (0.3–1.2)
**Age**	**≥40 years**	43	Non-linear	2.3 (1.4–3.2)	2.0 (0.9–3.2)	1.3 (-0.4–3.0)
	**<40 years**	37	Linear	0.9 (0.5–1.4)	0.9 (0.5–1.4)	0.9 (0.5–1.4)
**Symptom duration**	**≥10 years**	52	Non-linear	2.3 (1.4–3.1)	2.0 (0.9–3.0)	1.3 (-0.4–2.8)
	**<10 years**	25	Linear	0.6 (0.2–1.0)	0.6 (0.2–1.0)	0.6 (0.2–1.0)
**BMI**	**≥25 kg/m**^**2**^	26	Non-linear	2.5 (1.4–3.8)	2.1 (1.0–3.6)	0.9 (0.0–3.1)
	**<25 kg/m**^**2**^	22	Linear	0.9 (0.3–1.5)	0.9 (0.3–1.5)	0.9 (0.3–1.5)

Values are presented as mean (95% CI).

mSASSS: modified Stoke AS Spine Score; BMI: body mass index.

The majority (>65%) of patients with syndesmophytes at baseline had one or more other risk factors, e.g. male gender, age ≥40 years, symptom duration ≥10 years, and/or BMI ≥25kg/m^2^. A second risk factor in addition to the presence of syndesmophytes resulted in an additional increase in the progression rate of 0.2–0.4 mSASSS units during the first 2 years of follow-up (Table 4). Overall, progression rates reduced from maximal 3.2 mSASSS units over 0–2 years to minimal 1.2 mSASSS units over 4–6 years in patients with multiple risk factors (Table 4).

**Table 4:** GEE estimated mean 2-year spinal radiographic progression rates over time of AS patients with multiple risk factors.

	n	Course of progression	mSASSS progression rates		
			0–2 year (95% CI)	2–4 year (95% CI)	4–6 year (95% CI)
**Syndesmophytes**	43	Non-linear	2.8 (1.9–3.7)	2.5 (1.3–3.6)	1.6 (-0.1–3.3)
**Syndesmophytes & male gender**	35	Non-linear	3.0 (1.9–4.0)	2.7 (1.3–4.0)	1.8 (-0.2–3.9)
**Syndesmophytes & age ≥40 years**	31	Non-linear	3.0 (1.8–4.1)	2.6 (1.2–4.1)	1.6 (-0.6–4.1)
**Syndesmophytes & symptom duration ≥10 years**	35	Non-linear	3.1 (2.0–4.1)	2.7 (1.4–4.0)	1.6 (-0.4–3.6)
**Syndesmophytes & BMI ≥25 kg/m**^**2**^	19	Non-linear	3.2 (1.6–4.8)	2.6 (0.8–4.5)	1.2 (-1.6–3.9)

Values are presented as mean (95% CI). See Table 1 for abbreviations.

Complete case analysis in 53 AS patients with radiographic data available at all 2-year time points revealed similar results. An additional finding was that patients with a time since diagnosis of ≥5 years and current smokers also showed high but reducing progression rates over time (S3 Table).

### Radiographic progression in patients without risk factors

Patients without baseline syndesmophytes, female patients, patients age <40 years, symptom duration <10 years, and BMI <25kg/m^2^ showed less spinal damage at baseline (Table 3). Longitudinal modeling in these patients revealed that the course of spinal radiographic progression was linear with low estimated progression rates of ≤1 mSASSS units per 2 years. The lowest progression rates were found in patients without baseline syndesmophytes (Table 3, Fig 1).

Complete case analysis in 53 AS patients with radiographic data available at all 2-year time points revealed similar results (S3 Table).

## Discussion

In the present analysis, we evaluated the influence of patient characteristics on the long-term course of spinal radiographic progression in AS patients receiving prolonged TNF-α inhibitors in daily clinical practice. A small decrease in mSASSS progression rates during 6 years of follow up was observed in patients with risk factors for poor radiographic outcome, including the most important and well-known risk factor; baseline syndesmophytes. Furthermore, slow and linear progression was observed in patients without risk factors for poor radiographic outcome.

The decreasing progression rates might be a result of the stable low disease activity in patients receiving TNF-α inhibitors for a long period of time. In patients with baseline syndesmophytes, the 2-year progression rate nearly halved during the last 2-years of follow-up (4–6 years) as compared to the first 2 years of follow-up (0–2 years). Reducing progression rates were also found in male patients, patients with older age, longer symptom duration, and higher BMI. Very low and stable progression rates of ≤1 mSASSS units per 2 years were found in patients without these risk factors. A recent prospective longitudinal cohort study in 334 AS patients found that patients starting TNF-α inhibitors more than 10 years from start of symptoms had an increased risk (OR: 2.4) for radiographic progression compared to patients with a shorter delay, after correcting for baseline syndesmophytes. The authors indicated that the effect of TNF-α inhibitors on radiographic progression is most pronounced at an early stage of the disease, before structural damage is present.[7] The reducing progression rates in AS patients with more longstanding and advanced disease, as shown in the present study, suggest that TNF-α inhibitors may also influence spinal radiographic progression at a later stage of the disease.

Associations between radiographic progression and clinical disease activity, inflammatory markers, physical functioning, and NSAID use, as found in previous studies [5–7,10,16], could not be demonstrated in the present study. At least, variation in disease activity between patients over time is needed to show any possible association between disease activity and spinal radiographic progression.[11] Almost all patients had high disease activity at baseline and stable, low disease activity over time due to the effect of TNF-α inhibitors. As a consequence, no inter-patient variation of disease activity was present which explains that no significant association between disease activity and spinal radiographic progression could be found. The same explanation can be applied for the lack of association between NSAIDs and spinal radiographic progression in our study, since the use of NSAIDs was also high at baseline and decreased rapidly after starting TNF-α inhibitors. No differences in radiographic outcome were found in patients treated with the receptor antagonist (etanercept) and monoclonal antibodies (infliximab and adalimumab). However, these results should be taken with caution due to small numbers and the possibility of selection bias.

It is well-known that a healthy lifestyle is important to enhance an overall general health. Obesity and smoking are related to comorbidities, e.g. cardio-vascular diseases, diabetes, different forms of cancer, and increased mortality.[17,18] In the present study, we demonstrated that BMI is associated with worse radiographic outcome in AS. Overweight and obese patients (BMI ≥25kg/m^2^) had higher radiographic damage at baseline and an increased risk for developing radiographic damage over time. A comparable association has been found in a retrospective study in 47 AS patients with unknown follow-up duration.[19] Although BMI was not an independent risk factor, it may have a contributory role in the development of new bone in AS. In a SpA mouse model, new bone formation was associated with biomechanical stress at the entheseal sites and inflammation.[20] Increased body weight results in increased biomechanical stress. In addition, previous studies have found that BMI is associated with higher disease activity, including elevated CRP levels.[21,22] Less effect of TNF-α inhibitors on disease activity and physical functioning has been found in obese or overweight axSpA and AS patients.[23,24] Additional analysis in the present study also showed higher disease activity over time in patients with overweight or obesity (data not shown).

In addition to higher BMI, current smoking status was significantly associated with worse radiographic outcome in complete case analysis. This risk factor has also been reported in previous studies in AS and axial SpA.[6,7] From literature, it is known that smoking is associated with functional impairment, higher disease activity and more inflammation on MRI.[25,26] An observational cohort study in 1576 Danish AS patient showed that current and previous smokers had worse treatment response to TNF-α inhibitors than never smokers.[27] Components of tobacco smoke can activate immune responses leading to higher secretion of pro-inflammatory cytokines. More inflammation and functional impairment may result in more new bone formation.[6] Therefore, lifestyle coping such as weight reduction and smoking cessation should be taken into account during the management of AS.

During the analyses of long-term prospective data, losses to follow-up and incomplete follow-up data should be taken into account. The number of drop-outs in our cohort over 6 years of follow-up was rather low (12%) but we had to deal with missing data at intermediate follow-up visits (15% of all visits). We resolved this by using a single linear imputation technique in which a linear course was assumed during the imputed time intervals. Importantly, baseline characteristics were comparable between patients with complete and incomplete radiographic data. Complete case analysis revealed similar results as the analyses in the total group.

The mean estimated progression rates as presented in this study were derived from the GEE model with the best fit for the data. Significant contribution and goodness-of-fit of different time models were explored in order to investigate whether spinal radiographic progression followed a linear or non-linear course. Similar methodology has previously been used in the OASIS cohort and in our previous study to investigate the course of spinal radiographic progression and longitudinal relationships between variables.[3,4] Some caution is needed during the interpretation of the results in the small subgroups, such as the BMI groups in complete case analysis. Unfortunately, the measurement error of the mSASSS is relatively large, as was also demonstrated in other studies.[3] In our study the smallest detectable change was 2.3 for scoring 2-year mSASSS progression. This indicates that, on average, most patients and especially the patients without risk factors showed progression rates within the measurement error. Therefore, it is difficult to interpret whether these patients showed ‘real’ progression or whether the observed progression rate was caused by the measurement error.

In conclusion, the present study was the first that investigated the influence of important patient characteristics on the course of spinal radiographic progression in AS patients during long-term treatment with TNF-α inhibitors. AS patients at risk of developing radiographic damage showed a deflection from a linear course with reducing progression rates over time. The strongest deflection was found in patients with multiple risk factors, the presence of baseline syndesmophytes was the most important. In contrast, linear and very low progression rates, even smaller than measurement error, were observed in patients without risk factors, including patients without baseline syndesmophytes, female patients, non-smokers, and patients at younger age, with shorter disease duration, and normal BMI.

The data presented in this study are of great clinical importance since knowledge about the course of spinal radiographic progression in different patient groups will influence treatment decisions in daily clinical practice of AS. In addition, our results emphasize that clinicians and patients should be aware of the possible negative consequences of poor lifestyle factors such as overweight/obesity and smoking on radiographic outcome. Future studies conducted in well-organized large prospective cohorts with standardized follow-up visits are needed to confirm our results.

## Supporting information

S1 Table Baseline characteristics of AS patients with and without complete radiographic data at all 2-year time points during 6 years of follow-up. (DOCX)

S2 Table Associations between baseline characteristics and spinal radiographic damage over time in patients with complete radiographic data (n = 53). (DOCX)

S3 Table GEE estimated mean 2-year spinal radiographic progression rates of AS patients with complete radiographic data (n = 53), stratified for baseline risk factors. (DOCX)

S1 Fig Disease activity assessed with BASDAI and ASDAS in AS patients with 6 years of follow-up (n = 80). (DOCX)
