# Supplementary material for: Ankylosing spondylitis patients at risk of poor radiographic outcome show diminishing spinal radiographic progression during long-term treatment with TNF-α inhibitors
Source: PLoS One. 2017 Jun 22;12(6):e0177231. doi: 10.1371/journal.pone.0177231 (PMC5480831; doi:10.1371/journal.pone.0177231)
Supplement: S1 Table — (DOCX) [file pone.0177231.s001.docx]

**S1 Table.** Baseline characteristics of AS patients with and without complete radiographic data at all 2-year time points during 6 years of follow-up.

|  | **Complete radiographic data** | **Missing radiographic data** |  |
| --- | --- | --- | --- |
|  | **n=53** | **n=27** | **p-value** |
| **Male gender** | 38 (72) | 18 (67) | 0.642 |
| **Age (yrs)** | 40.2 ± 11.1 | 43.3 ± 9.0 | 0.218 |
| **Symptom duration (yrs)** | 15 (7-21) | 13 (8-26) | 0.315 |
| **Time since diagnosis (yrs)** | 5 (1-14) | 7 (1-17) | 0.802 |
| **HLA-B27+** | 43 (81) | 19 (70) | 0.276 |
| **BMI (kg/m^2^)** | 25.3 ± 3.8 | 26.2 ± 3.7 | 0.413 |
| **Current smoker** | 17 (37) | 11 (52) | 0.235 |
| **Smoking duration (yrs)** | 13 (0-26) | 14 (6-26) | 0.763 |
| **NSAID use** | 45 (85) | 24 (89) | 0.742 |
| **ASAS-NSAID index** | 67 (38-100) | 50 (25-100) | 0.167 |
| **DMARD use** | 16 (30) | 5 (19) | 0.262 |
| **BASDAI (0-10)** | 5.8 ± 1.7 | 6.5 ± 1.7 | 0.076 |
| **ASDAS_CRP_** | 3.8 ± 0.8 | 3.9 ± 0.8 | 0.457 |
| **Patient’s GDA (0-10)** | 7 (5-8) | 7 (5-8) | 0.440 |
| **CRP (mg/L)** | 14 (7-25) | 13 (7-21) | 0.638 |
| **BASFI (0-10)** | 5.3 (3.5-7.0) | 6.0 (4.1-7.2) | 0.348 |
| **mSASSS (range 0-72) mean** | 8.2 ± 12.9 | 9.6 ± 14.3 |  |
| **median** | 3.7 (0.0-11.4) | 3.0 (0.0-12.3) | 0.757 |
| **≥1 syndesmophyte** | 28 (53) | 15 (56) | 0.817 |

Values are presented as number of patients (%), mean ± SD, or median (IQR).

AS: ankylosing spondylitis; HLA: human leukocyte antigen; BMI: body mass index; NSAID: non-steroidal anti-inflammatory drug; ASAS: Assessment of SpondyloArthritis international Society; DMARD: disease-modifying anti-rheumatic drug; BASDAI: Bath AS disease activity index; ASDAS: AS disease activity score; GDA: global disease activity; BASFI: Bath AS functional index; CRP: C-reactive protein; mSASSS: modified Stoke AS spine score.
