# Supplementary material for: Ankylosing spondylitis patients at risk of poor radiographic outcome show diminishing spinal radiographic progression during long-term treatment with TNF-α inhibitors
Source: PLoS One. 2017 Jun 22;12(6):e0177231. doi: 10.1371/journal.pone.0177231 (PMC5480831; doi:10.1371/journal.pone.0177231)
Supplement: S2 Table — (DOCX) [file pone.0177231.s002.docx]

**S2 Table.** Associations between baseline characteristics and spinal radiographic damage over time in patients with complete radiographic data (n=53).

|  | **Complete cases** | |
| --- | --- | --- |
|  | **B (95% CI)** | **p-value** |
| **Male gender** | 7.95 (0.98-14.91) | **0.025** |
| **Age (yrs)** | 0.71 (0.35-1.07) | **<0.001** |
| Age **≥**40 years | 11.37 (3.91-18.83) | **0.003** |
| **Symptom duration (yrs)** | 1.04 (0.59-1.50) | **<0.001** |
| Symptom **≥**10 years | 12.83 (6.79-18.87) | **<0.001** |
| **Time since diagnosis (yrs)** | 1.06 (0.42-1.71) | **0.001** |
| Time since diagnosis **≥**5 years | 10.79 (4.00-17.57) | **0.002** |
| **HLA-B27+** | -4.08 (-10.29-2.14) | 0.199 |
| **Current smoker** | 9.11 (0.11-18.11) | **0.047** |
| **Smoking duration** | 0.27 (-0.09-0.63) | 0.147 |
| **BMI (kg/m^2^)** | 1.82 (0.08-3.55) | **0.040** |
| BMI ≥25 kg/m^2^ | 13.15 (1.80-24.50) | **0.023** |
| **NSAID use** | 1.31 (8.25-10.87) | 0.788 |
| **ASAS-NSAID index** | -0.02 (-0.11-0.08) | 0.737 |
| **DMARD use** | 0.24 (-8.74-9.22) | 0.958 |
| **First TNF-α inhibitor†** | -8.18 (-17.71-1.35) | 0.093 |
| **BASDAI (0-10)** | -0.55 (-2.21-1.11) | 0.518 |
| **ASDAS_CRP_** | 1.07 (-3.65-5.79) | 0.656 |
| **Patient’s GDA (0-10)** | 0.08 (-1.36-1.52) | 0.916 |
| **CRP (mg/L)** | -0.03 (-0.27-0.22) | 0.845 |
| **BASFI (0-10)** | 0.96 (-0.54-2.46) | 0.209 |
| **mSASSS** | 1.12 (1.02-1.22) | **<0.001** |
| **≥1 syndesmophyte** | 18.25 (12.51-23.99) | **<0.001** |

Values are presented as number of patients (%), mean ± SD, or median (IQR).

AS: ankylosing spondylitis; HLA: human leukocyte antigen; BMI: body mass index; NSAID: non-steroidal anti-inflammatory drug; ASAS: Assessment of SpondyloArthritis international Society; DMARD: disease-modifying anti-rheumatic drug; BASDAI: Bath AS disease activity index; ASDAS: AS disease activity score; GDA: global disease activity; BASFI: Bath AS functional index; CRP: C-reactive protein; mSASSS: modified Stoke AS spine score.

†Etanercept vs. infliximab/adalimumab
