# Supplementary material for: Ankylosing spondylitis patients at risk of poor radiographic outcome show diminishing spinal radiographic progression during long-term treatment with TNF-α inhibitors
Source: PLoS One. 2017 Jun 22;12(6):e0177231. doi: 10.1371/journal.pone.0177231 (PMC5480831; doi:10.1371/journal.pone.0177231)
Supplement: S3 Table — (DOCX) [file pone.0177231.s003.docx]

**S3 Table.** GEE estimated mean 2-year spinal radiographic progression rates of AS patients with complete radiographic data (n=53), stratified for baseline risk factors.

|  |  | **n** | **Course of progression** | **Estimated mSASSS progression rates** | | |
| --- | --- | --- | --- | --- | --- | --- |
|  |  |  |  | **0-2 year** | **2-4 year** | **4-6 year** |
| **Total group** |  | 53 | Non-linear | 1.8 | 1.6 | 1.1 |
| **Syndesmophytes** | **Yes** | 28 | Non-linear | 3.0 | 2.7 | 1.7 |
|  | **No** | 25 | Linear | 0.4 | 0.4 | 0.4 |
| **Gender** | **Male** | 38 | Non-linear | 2.2 | 1.9 | 1.2 |
|  | **Female** | 15 | Linear | 0.7 | 0.7 | 0.7 |
| **Age** | **≥40 years** | 25 | Non-linear | 2.6 | 2.3 | 1.4 |
|  | **<40 years** | 28 | Linear | 0.9 | 0.9 | 0.9 |
| **Symptom duration** | **≥10 years** | 32 | Non-linear | 2.6 | 2.2 | 1.3 |
|  | **<10 years** | 18 | Linear | 0.6 | 0.6 | 0.6 |
| **Time since diagnosis** | **≥5 years** | 29 | Non-linear | 2.4 | 2.1 | 1.2 |
|  | **<5 years** | 24 | Linear | 0.9 | 0.9 | 0.9 |
| **Current smoker** | **Yes** | 17 | Non-linear | 2.8 | 1.8 | 1.3 |
|  | **No** | 29 | Linear | 1.2 | 1.2 | 1.2 |
| **BMI** | **≥25 kg/m^2^** | 15 | Non-linear | 3.0 | 2.6 | 1.2 |
|  | **<25 kg/m^2^** | 13 | Linear | 1.1 | 1.1 | 1.1 |

Values are presented as mean ± standard deviation.

GEE: Generalized Estimation Equations; AS: ankylosing spondylitis; mSASSS: modified Stoke AS spine score; BMI: body mass index.
