# Supplementary material for: Ankylosing spondylitis patients at risk of poor radiographic outcome show diminishing spinal radiographic progression during long-term treatment with TNF-α inhibitors
Source: PLoS One. 2017 Jun 22;12(6):e0177231. doi: 10.1371/journal.pone.0177231 (PMC5480831; doi:10.1371/journal.pone.0177231)
Supplement: S1 Fig — (DOCX) [file pone.0177231.s004.docx]

**S1 Figure.** Disease activity assessed with BASDAI and ASDAS in AS patients with 6 years of follow-up (n=80).

**
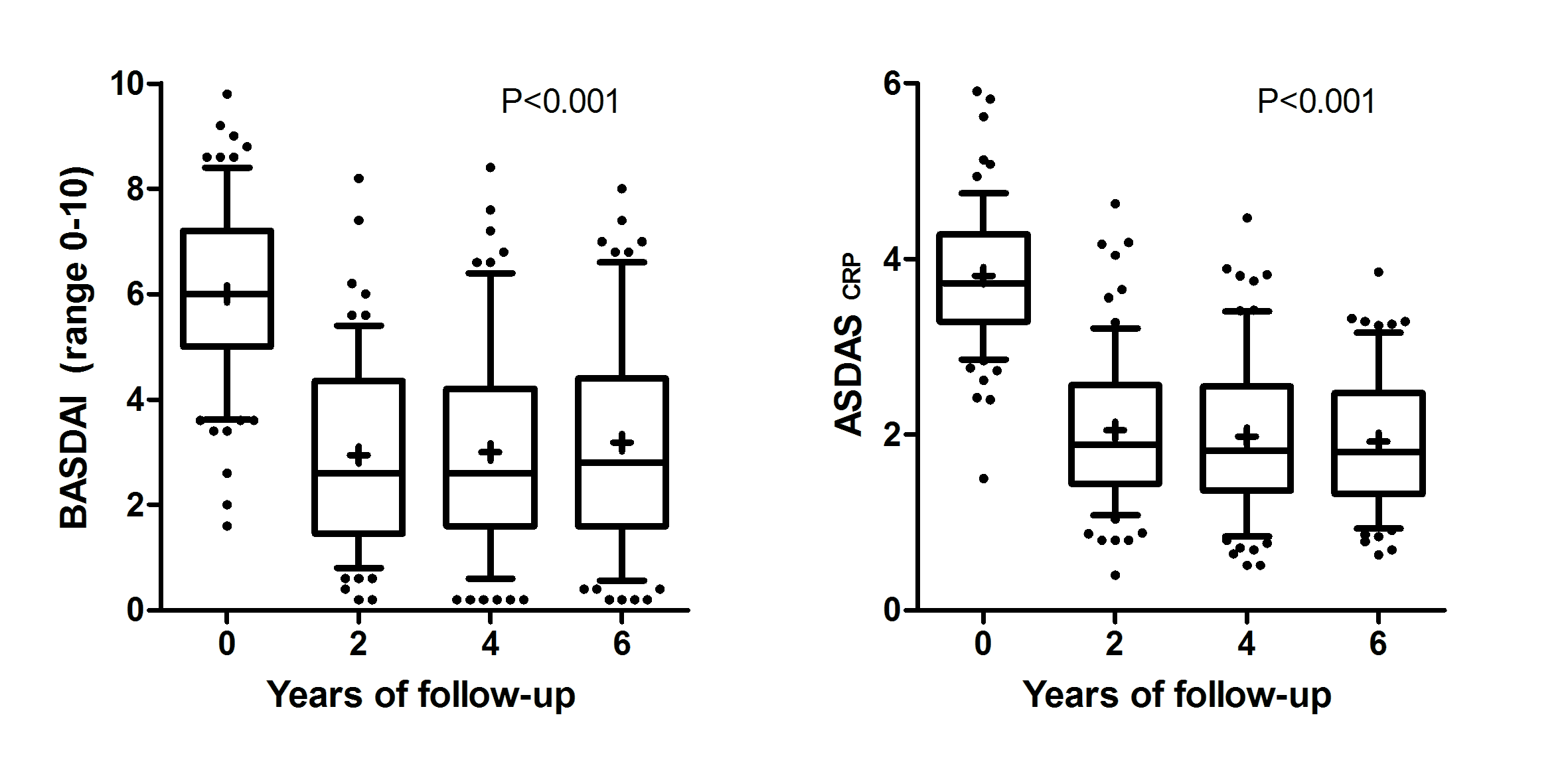
**
